# Impact of human papillomavirus vaccine on cervical cancer epidemic: Evidence from the surveillance, epidemiology, and end results program

**DOI:** 10.3389/fpubh.2022.998174

**Published:** 2023-01-06

**Authors:** Jiao Pei, Ting Shu, Chenyao Wu, Mandi Li, Minghan Xu, Min Jiang, Cairong Zhu

**Affiliations:** ^1^Department of Epidemiology and Health Statistics, West China School of Public Health and West China Fourth Hospital, Sichuan University, Chengdu, China; ^2^Sichuan Cancer Hospital & Institute, Sichuan Cancer Center, School of Medicine, University of Electronic Science and Technology of China, Chengdu, China; ^3^The Healthcare Department, Chengdu Women's and Children's Central Hospital, School of Medicine, University of Electronic Science and Technology of China, Chengdu, China

**Keywords:** HPV vaccine, cervical cancer, incidence, projection, vaccine impact

## Abstract

**Introduction:**

Since 2006, the human papillomavirus (HPV) vaccine has been recommended for females aged 9–26 years in the United States. Aiming to evaluate the early effect of the HPV vaccine on cervical cancer, this study assessed the incidence of cervical cancer by age and histology before and after the introduction of HPV vaccination.

**Methods:**

Data on cervical cancer incidence from 1975–2019 were extracted from the Surveillance, Epidemiology, and End Results Program. Joinpoint regression was used to determine temporal trends over time. Future cervical cancer incidence (2015–2039) was projected using Bayesian age-period-cohort analysis. Age-period-cohort (APC) models were created to evaluate age, period, and cohort effects.

**Results:**

For overall cervical cancer and cervical squamous cell carcinoma (SCC), incidence rate showed decreasing trends (–0.7%, and –1.0% annually, respectively), whereas cervical adenocarcinoma (AC) incidence continuously increased (2.6% annually). The incidence trends for AC were stable in the 20–24 and 25–29-year age groups, whereas there was an increasing trend in older age groups. Similarly, the projected trend for AC in females aged 20–30 years exhibited a decline, whereas an increase was predicted in the 31–40–year age group, especially in the 35–44 year age group. The birth cohort and period effects in SCC and AC were extracted from APC models.

**Discussion:**

During the period of 1975–2019, the incidence of cervical AC remained almost unchanged in the age groups receiving HPV vaccines while increased in the age groups not receiving HPV vaccines. The birth cohort effects of SCC and AC of the cervix provided evidence supporting the effectiveness of the HPV vaccine in preventing cervical cancer.

## Introduction

Cancer of the uterine cervix ranks as the fourth most common cancer worldwide among females accounting for 604 127 cases and 341 831 deaths in 2020 (1). It is estimated that up to 14,100 and 4,280 new invasive cervical cancer cases and deaths, respectively, will be diagnosed in the United States (US) in 2022 (2). The primary etiologic factor for cervical cancer is persistent human papillomavirus (HPV) infection (3). Bivalent/quadrivalent/9-valent HPV vaccines have been recommended for females aged 9–26 years by the US Advisory Committee on Immunization Practices since 2009 (4), 2006 (5), and 2015 (6), respectively. The bivalent vaccine (2vHPV) and quadrivalent vaccine (4vHPV) are directed against two oncogenic types (HPV 16 and 18) (4), which are responsible for ~70% of cervical cancers (7). Quadrivalent vaccine (4vHPV) covers other two low-risk HPV genotypes (HPV 6 and 11), which account for 90% of anogenital warts (8). The 9-valent vaccine (9vHPV) covers the other five oncogenic types (HPV 31, 33, 45, 52, and 58) that are responsible for another 20% of cervical cancers (7). In the US, the uptake of ≥1 dose of HPV vaccines increased from 37.2 to 69.9% from 2008 to 2018 among adolescent females aged 13–17 years, compared to 20.7–52.8% from 2010 to 2018 among females aged 19–26 years (9–11). Meanwhile, the proportion of female teens who completed the full series of HPV vaccination increased from 17.9% in 2008 to 53.7% in 2018 (9, 10).

Some studies investigated the vaccine's impact on HPV-associated health outcomes (7, 12–22); of these, the majority focused on the effect in reducing the prevalence of the HPV infection and high-grade cervical lesions (12–22). To date, only one study assessed trends in cervical cancer incidence after the introduction of HPV vaccination among young females in the US (7). However, that study did not comprehensively examine the birth cohort effects before and after initiation of HPV vaccination decades ago, nor did it predict future trends of cervical cancer incidence. Among sexually active females aged 14–24 years who participated in the National Health and Nutrition Examination Survey (NHANES), the rate of 4vHPv (types 6/11/16/18) prevalence was reduced compared to that between 2003 and 2006 (15). Previous modeling suggested that the full effect of HPV vaccination in this age group would not be observed until 2025, but half of the benefit would be seen in England by 2019, where the HPV vaccine was first introduced in 2008 (23). The association between quadrivalent/bivalent HPV vaccination and the risk of invasive cervical cancer was assessed in 2020 (24) and 2021(25), respectively. Data used in the studies was retrieved from nationwide Swedish demographic and health registers from 2006 to 2017 (24) and population-based cancer registry in England from 2006 to 2019 (25). The HPV vaccine was introduced in the US 13 years ago, and although it may be premature to assess its full benefits, its early impact can be studied by considering the incidence trends of cervical cancer. To better evaluate the early impact of the HPV vaccine on cervical cancer, we described temporal trends of cervical cancer incidence by age and histology, and built age-period-cohort (APC) models to estimate period, and birth cohort effects, before and after the introduction of HPV vaccination among females aged 20–44 years, using data from the Surveillance, Epidemiology, and End Results (SEER) Program. The period effects referred to policy and environmental factors that impact the populations of all ages. The cohort effects were due to historical factors on certain populations born in the same era. Further, we also projected the cervical cancer incidence by age and histology to 2,039 based on the HPV vaccine introduction by performing Bayesian age-period-cohort (BAPC) models that incorporate age, calendar period, and birth cohort effects.

## Materials and methods

### Data sources

The data of cervical cancer incidence for 1975–2019 was extracted from the Surveillance, Epidemiology, and End Results (SEER) Program: SEER 9 registries for 1975–1991, which cover 9.4% of the total US population (26), SEER 13 registries for 1992–1999, which cover 13.4% of the US population (26), and SEER 22 registries for 2000–2019, which covered 47.9% of the US population (27). The SEER Program is an authoritative source for cancer statistics in the United States as it is supported by the Surveillance Research Program (SRP) from the NCI's Division of Cancer Control and Population Sciences (DCCPS). The SEER data used here was released in November 2020 and November 2021 [SEER^*^Stat Database: Incidence—SEER 9, Nov 2020 Sub (1975–2018), SEER 13, Nov 2020 Sub (1992–2018), SEER 22, Nov 2021 Sub (2000–2019), Single Ages to 85+]. Cervical cancer data was divided into 5 age groups (20–24, 25–29, 30–34, 35–39, and 40–44 years) and was identified as site record ‘Cervix Uteri’ according to the International Classification of Diseases for Oncology, Third Edition (ICD-O-3), with histology codes of 8000-9992. Cervical cancer cases are classified into four histologic subtypes: squamous cell carcinoma (SCC) (ICD-O-3 8050-8130), adenocarcinoma (AC) (ICD-O-3 8140-8147, 8160-8162, 8180-8221, 8250-8506, 8520-8550, 8570-8573, 8940-8941), adenosquamous carcinoma (ICD-O-3 8560-8563), and others (including cervical cancers with unknown histology or unspecified carcinomas). Due to sparse data, cervical adenosquamous carcinoma and other histologic subtypes were excluded from subgroup analysis of this study.

A total of 160 868 newly diagnosed cases of cervical cancer were reported to SEER 9, SEER 13, and SEER 22 programs from 1975 to 2019, with 108 364 (67.36%) cases of SCC and 32 523 (20.22%) of AC. The SCC and AC types accounted for 74.43% and 8.86% of cervical cancers in 1975, whereas they accounted for 64.32% and 24.92% of cases in 2019, respectively.

### Description of temporal trends

The incidence rates, for SCC, AC of the cervix, and all histopathologic subtypes combined were calculated from 1975 to 2019 for the 20–44-year-old group. In order to describe the incidence trends by birth cohort group, the majority of females aged 15–64 years were included to analyze considering the few cohort groups of females aged 20–44. Herein, there were ten cohort groups (1948–1952, 1953–1957, 1958–1962, 1963–1967, 1968–1972, 1973–1977, 1978–1982, 1983–1987, 1988–1992, and 1993–1997) in this section. The logarithmic transformation of rates in cervical cancer incidence by histology were used to show the pace of changes of incidence trends.

### Joinpoint regression model

The trends in the incidence rates of cervical cancer during 1975–2019 by age group and histological subtype were depicted using joinpoint regression analyses, which provided annual percent changes (*APC*s) and average annual percent changes (*AAPC*s) (28). Joinpoint regression models were fitted to identify the joinpoints (year) when annual percentage changes (*APC*s) changed significantly. Annual percentage changes (*APC*s) were used to characterize the magnitude and direction of trends. It was calculated as {exp(β)−1} × 100, where the regression coefficient (β) was estimated by fitting a least squares regression line to a logarithmic transformation of incidence rates, using the calendar year as an independent variable. Joinpoint regression uses least squares regression to fit line segments to the log-scale rates, joined at discrete points that represent statistically significant changes in direction of the trend. Average annual percent change (*AAPC*) is a summary measure of the trend over a pre-specified fixed interval. It allows us to use a single number to describe the average *APC*s over a period of multiple years (28). We specified a logarithmic transformation of incidence rates and a maximum number of three joinpoints to avoid capturing unstable trends due to relatively small numbers of cases in some age groups.

### Age-period-cohort model

Age-period-cohort (APC) models were built to evaluate age, period, and birth cohort effects for cervical cancer based on five 5-year age groups (20–24, 25–29, 30–34, 35–39, and 40–44 years old) and nine 5-year time periods (1975–1979, 1980–1984, 1985–1989, 1990–1994, 1995–1999, 2000–2004, 2005–2009, 2010–2014, and 2015–2019). The age effects are the changes in disease rate with age, independent of birth cohort effects and period effects, on account of risk factors linked to maturation. The period effects are caused by factors impacting all subjects during a particular period, such as the implementation of a cervical cancer screening policy. The cohort effects refer to historical factors on certain populations born in the same era, such as the HPV vaccine initiation for younger age groups which was not available for older generations. Assuming to use a log-linear Poisson regression model, the APC model is:


$$\begin{array}{l} \ln\left(\frac{y_{ij}}{n_{ij}}\right)=\mu +\alpha_{i}+\beta_{j}+\gamma_{k} \end{array}$$

where *y*_*ij*_ represents the number of cases, and *n*_*ij*_ the population. The parameter α_*i*_ represents the age group *i* effect, β_*j*_ the time period *j* effect, and γ_*k*_ the birth cohort *k* effect. Due to the significant linear correlation among age, period, and birth cohort [*period*(*P*) − *age*(*A*) = *cohort*(*C*)], the APC model presented the non-identifiability issue, i.e., the independent effects of age, period, and cohort cannot be evaluated simultaneously. However, age and birth cohort effects could be estimated by constraining the slope of period effect to be 0 (β_*j*_ = 0) (29). To verify the cohort effects, we performed sensitivity analyses under different assumptions regarding the slope of the period effect, β_p_ = – 0.01 or + 0.01, which indicated that the period slope decreased or increased, respectively. Moreover, goodness of fit of the APC models was examined using residual deviance statistics by Clayton and Schiffler approach (30–32). The linear time variations are referred to as a parameter “drift” which can be partitioned into any linear combination of period and cohort effects. Therefore, the overall sub-models of the APC model by Clayton and Schiffler are as follows: ① age-only model (A), ② age-drift model, ③ age-cohort model (AC), ④ age-period model (AP), and ⑤ age-period-cohort model. Each sub-model fit was assessed by comparing each iterative model with sequentially adding period and cohort effects to the primary model of age alone to determine whether these added parameters significantly improved model fit. Significant differences were tested in residual deviance of each pairwise comparison using chi-squared tests.

### Bayesian age-period-cohort model

Future age-specific cervical cancer incidence and cases from 2015 to 2039 by histology were projected using the Bayesian age-period-cohort analysis (BAPC) with integrated nested Laplace approximations (INLA) (33), which was proved to be the only method to achieve reasonable projections (34). BAPC models involve no parametric assumptions, expecting that all parameters are from appropriate prior distributions. Commonly, smoothing priors, i.e., first-order random walk (RW1) and second-order random walk (RW2), are used for age, period, and birth cohort effects when the assumption that effects adjacent in time might be similar (35). The RW2 prior assumes independent mean-zero normal distributions on the second differences of all time effects (34) and is suitable for a linear time trend, whereas the RW1 prior grants using constant extrapolation (33). Based on the APC model, with the addition of unstructured heterogeneity *z*_*ij*_, the Bayesian APC model can be expressed as


$$\begin{array}{l} \ln\left(\frac{y_{ij}}{n_{ij}}\right)=\mu +\alpha_{i}+\beta_{j}+\gamma_{k}+z_{ij} \end{array}$$

For *z*_*ij*_, Gaussian random effects $z_{ij}\sim N\left(0,\;\kappa_{z}^{-1}\right)$ can be added to the linear predictor $\ln\left(\frac{y_{ij}}{n_{ij}}\right)$ (33). The hyperprior of gamma distribution *k* ~ *G* (*a, b*) was assumed for all precision parameters with shape parameter *a* = 1 and rate parameter *b* = 0.005.

Calculations were restricted to individuals targeted by the US HPV vaccination program, including cases in 20–24 to 40–44 years age groups for each year between 1975 and 2019 and excluding females younger than 20 years with extremely low cervical cancer incidence. Adults ≥34 years old in 2017 had low vaccine uptake (2.7%) (36), so we conservatively assumed no association between vaccination and observed cervical cancer in 35–39 and 40–44 age groups in 2015–2019. We projected future cervical cancer incidence under the scenario of no HPV vaccination in 35–39 and 40–44 age groups, assuming that the observed contemporary trends of cervical cancer incidence continue (because the model that used current levels of HPV vaccination to estimate their future impact).

Information on cases was exported from SEER^*^Stat (version 8.4.0) to R (version 3.6.1) for further statistical analyses. Joinpoint Regression analyses were conducted using Joinpoint Desktop Software version 4.9.0.1 (National Cancer Institute, Bethesda, MD, USA). All APC and BAPC analyses were performed using the apc.fit function from the R-package Epi and the R-package BAPC, respectively. The BAPC package was built upon INLA-package (version 20.03.07), to predict future cancer rates and cases within a fully Bayesian inference setting. The national projected population was used for estimated population sizes in 2017 and beyond. All tests were 2-sided at a significance level of α = 0.05, with 95% confidence intervals.

Data used in this research are publicly available and are deidentified, hence this study was exempt from approval of the institutional review board.

## Results

A total of 63 304 cervical cancer cases in the 20–44 age groups were newly diagnosed from 1975 to 2019. Among them, the numbers of cervical SCC, cervical AC, cervical adenosquamous carcinoma and other histologic subtypes cases were 42 220 (66.69%), 13 694 (21.63%), 2 456 (3.88%), and 4 934 (7.79%), respectively.

**Figure 1:**
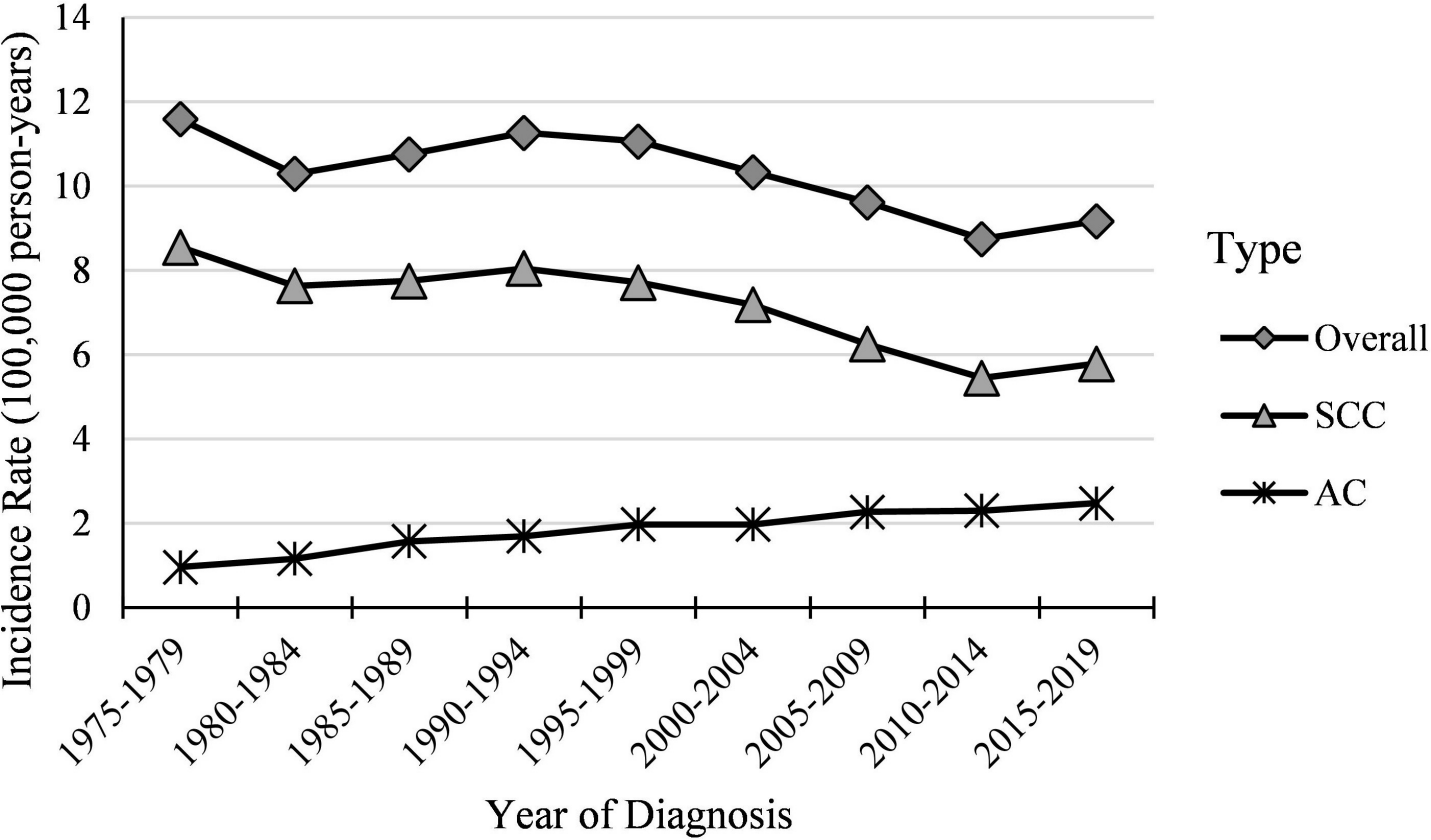
Temporal trends in cervical cancer incidence rates (1975–2019) among females aged 20 to 44 years by histology, United States. All data are expressed as the rate per 100,000.

**Figure 2:**
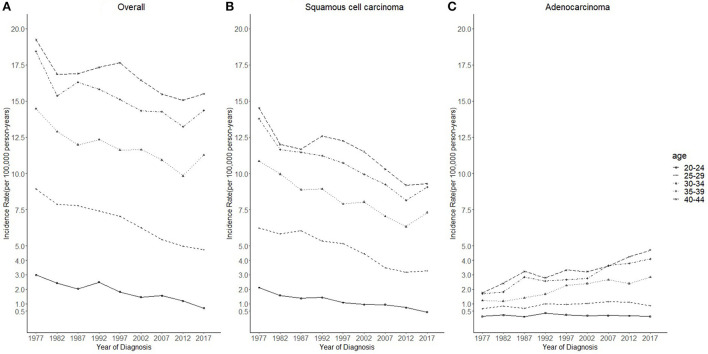
Temporal trends in cervical cancer incidence rates (1975–2019) among females aged 20 to 44 years by age and histology, United States. **(A)** Temporal trends of all histopathologic subtypes combined. **(B)** Temporal trends of squamous cell carcinoma (SCC). **(C)** Temporal trends of adenocarcinoma (AC). All data are expressed as the rate per 100,000.

**Figure 3:**
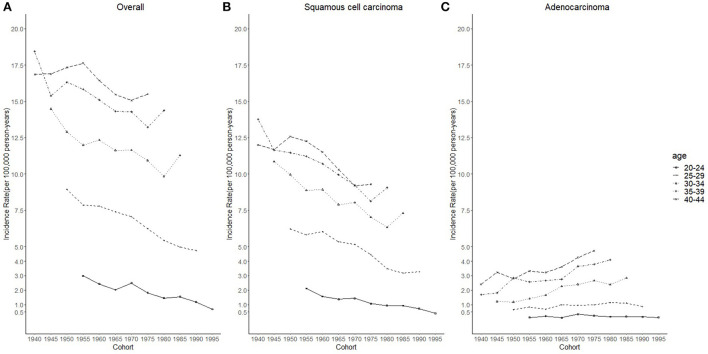
Cohort trends in cervical cancer incidence rates (1975–2019) among females aged 20 to 44 years by age and histology, United States. **(A)** Cohort trends of all histopathologic subtypes combined. **(B)** Cohort trends of squamous cell carcinoma (SCC). **(C)** Cohort trends of adenocarcinoma (AC). All data are expressed as the rate per 100,000.

Cases and rates on cervical cancer incidence (1975–2019) for females aged 20 to 44 years by year of diagnosis (i.e., time period) and age group were shown in Tables 1, 2, respectively. The incidence rates for cervical SCC, AC, and all histologic subtypes combined for the 20–44 years age group are presented in Figure 1. Similar to the overall histological incidence rate, the incidence rate of cervical SCC confirmed that cervical cancer generally decreased (overall: −0.7% per year, SCC: −1.0% per year), except for 1980–1994 or 2010–2019. However, the incidence rate of cervical AC continuously increased throughout 1975–2019 (2.6% annually). The trends of age-specific incidence rates over time period for SCC, AC, and all histological types combined according to age group are shown in Figure 2. *APC*s and *AAPC*s in the five age groups by histological subtype are presented in Table 3. For overall cervical cancer and SCC of the cervix, the increase during 2010–2019 was largely confined to ages 30–34, 35–39, and 40–44 years (Figures 2A,B). The trends for AC of the cervix were stable in the 20–24 and 25–29 age groups, and there was a distinct increase in older age groups (Figure 2C). The birth cohort trends of age-specific incidence rates for overall cervical cancer, SCC, and cervical AC by age groups are shown in Figure 3, which showed the trends over birth cohort The incidence rates for overall cervical cancer and SCC of the cervix reflected generally decreasing trends by cohort (Figures 3A,B). In contrast, except for a stable trend in cervical AC incidence rates in the 20–24 age group, the incidence visibly increased in the 30–44 age group and slightly increased in the 25–29 age group. It should be noted that in the 20–24 and 25–29-year groups, the 1983–1997 cohorts showed decreasing trends of incidence rates (Figure 3C).

**Table 1:** Cases and rates on cervical cancer incidence (1975–2019) for females aged 20–44 years by histology and year of diagnosis, United States.

**Year of diagnosis^b^**	**Person-years at risk**	**Overall**		**SCC**		**AC**	
		**Cases**	**Rate^a^**	**Cases**	**Rate^a^**	**Cases**	**Rate^a^**
1975–1979	19,140,808	2,217	11.583	1,636	8.547	184	0.961
1980–1984	21,728,878	2,236	10.290	1,659	7.635	253	1.164
1985–1989	23,647,795	2,543	10.754	1,832	7.747	373	1.577
1990–1994	32,002,733	3,605	11.265	2,574	8.043	543	1.697
1995–1999	36,964,306	4087	11.057	2,850	7.710	729	1.972
2000–2004	126,868,132	13,102	10.327	9,115	7.185	2,508	1.977
2005–2009	126,257,333	12,140	9.615	7,895	6.253	2,866	2.270
2010–2014	129,050,154	11,281	8.742	7,025	5.444	2,973	2.304
2015–2019	131,903,710	12,093	9.168	7,634	5.788	3,265	2.475

SCC, cervical squamous cell carcinoma; AC, cervical adenocarcinoma.

a Rate (per 100,000 person-years): equals to $\frac{\text{the number of cases}}{\text{person}-\text{years at risk}}\times 100,000$.

b Year of Diagnosis: time period.

**Table 2:** Cases and rates on cervical cancer incidence (1975–2019) for females aged 20–44 years by histology and age group, United States.

**Age group**	**Person-years at risk**	**Overall**		**SCC**		**AC**	
		**Cases**	**Rate^a^**	**Cases**	**Rate^a^**	**Cases**	**Rate^a^**
20–24	128,044,011	1,847	1.442	1,156	0.903	224	0.175
25–29	132,045,070	7,703	5.834	5,311	4.022	1,306	0.989
30–34	131,148,232	14,775	11.266	9,960	7.594	3,114	2.374
35–39	129,084,669	18,648	14.446	12,381	9.591	4,304	3.334
40–44	127,241,867	20,331	15.978	13412	10.541	4,746	3.730

SCC, cervical squamous cell carcinoma; AC, cervical adenocarcinoma.

a Rate (per 100,000 person-years): equals to $\frac{\text{the number of cases}}{\text{person}-\text{years at risk}}\times \;100,000$.

**Table 3:** Trends on cervical cancer incidence rates (1975–2019) for females aged 20–44 years by histology and age, United States.

	**Trends 1975–2019**									***AAPC*** **1975–2019**	
	**Trend 1**			**Trend 2**			**Trend 3**			
	**Time period**	***APC***	**95%CI**	**Time period**	***APC***	**95%CI**	**Time period**	***APC***	**95%CI**	
Overall										
20–24 years	1975–2011	−2.1^*^	(−2.7,−1.4)	2011–2019	−11.0^*^	(−16.3,−5.3)				−3.8^*^	(−4.9,−2.6)
25–29 years	1975–2019	−1.6^*^	(−1.8, −1.4)							−1.6^*^	(−1.8, −1.4)
30–34 years	1975–1978	−8.9	(−18.7, 2.0)	1978–2015	−0.7^*^	(−0.9, −0.4)	2015–2019	4.8	(−2.4, 12.6)	−0.8	(−1.8, 0.2)
35–39 years	1975–2019	−0.6^*^	(−0.8, −0.4)							−0.6^*^	(−0.8, −0.4)
40–44 years	1975–2019	−0.5^*^	(−0.7, −0.3)							−0.5^*^	(−0.7, −0.3)
SCC										
20–24 years	1975–2012	−2.5^*^	(−3.1, −1.8)	2012–2019	−13.2^*^	(−19.9, −6.0)				−4.3^*^	(−5.5, −3.0)
25–29 years	1975–2000	−0.9^*^	(−1.5, −0.2)	2000–2005	−7.3	(−16.5, 2.8)	2005–2019	−0.7	(−2.3, 0.8)	−1.6^*^	(−2.8, −0.3)
30–34 years	1975–2014	−1.4^*^	(−1.7, −1.2)	2014–2019	5.1	(−1.2, 11.7)				−0.7	(−1.4, 0.0)
35–39 years	1975–2019	−1.1^*^	(−1.3, −0.9)							−1.1^*^	(−1.3, −0.9)
40–44 years	1975–1982	−4.2^*^	(−6.8, −1.5)	1982–1996	0.6	(−0.5, 1.6)	1996–2019	−1.6^*^	(−2.0, −1.1)	−1.3^*^	(−1.9, −0.8)
AC										
20–24 years	1975–2019	13.5^*^	(3.6, 24.2)							13.5^*^	(3.6, 24.2)
25–29 years	1975–2010	2.0^*^	(1.0, 2.9)	2010–2019	−5.1	(−11.6, 1.9)				0.5	(−1.1, 2.1)
30–34 years	1975–2019	2.6^*^	(2.0, 3.1)							2.6^*^	(2.0, 3.1)
35–39 years	1975–2019	2.2^*^	(1.7, 2.6)							2.2^*^	(1.7, 2.6)
40–44 years	1975–2019	2.0^*^	(1.6, 2.5)							2.0^*^	(1.6, 2.5)

CI, confidence interval; APC, annual percent change; AAPC, average annual percent change; SCC, cervical squamous cell carcinoma; AC, cervical adenocarcinoma.

* Indicates that the APC or AAPC is significantly different from zero at the alpha = 0.05 level.

**Figure 4:**
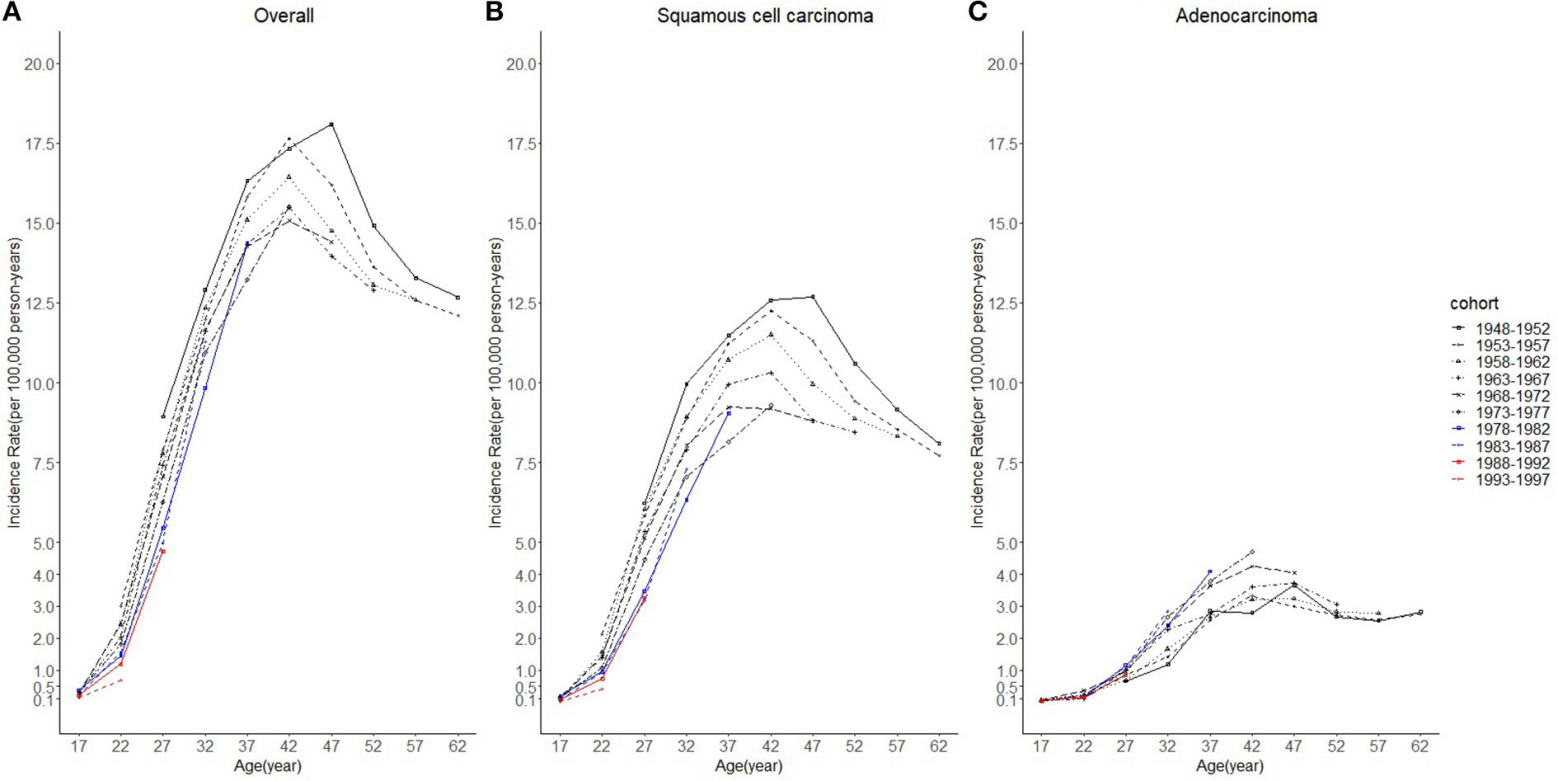
Age trends in cervical cancer incidence rates (1975-2019) among females in 1948-1997 birth cohorts by cohort and histology, United States. **(A)** Age trends of all histopathologic subtypes combined. **(B)** Age trends of squamous cell carcinoma (SCC). **(C)** Age trends of adenocarcinoma (AC). All data are expressed as the rate per 100,000.

**Figure 5:**
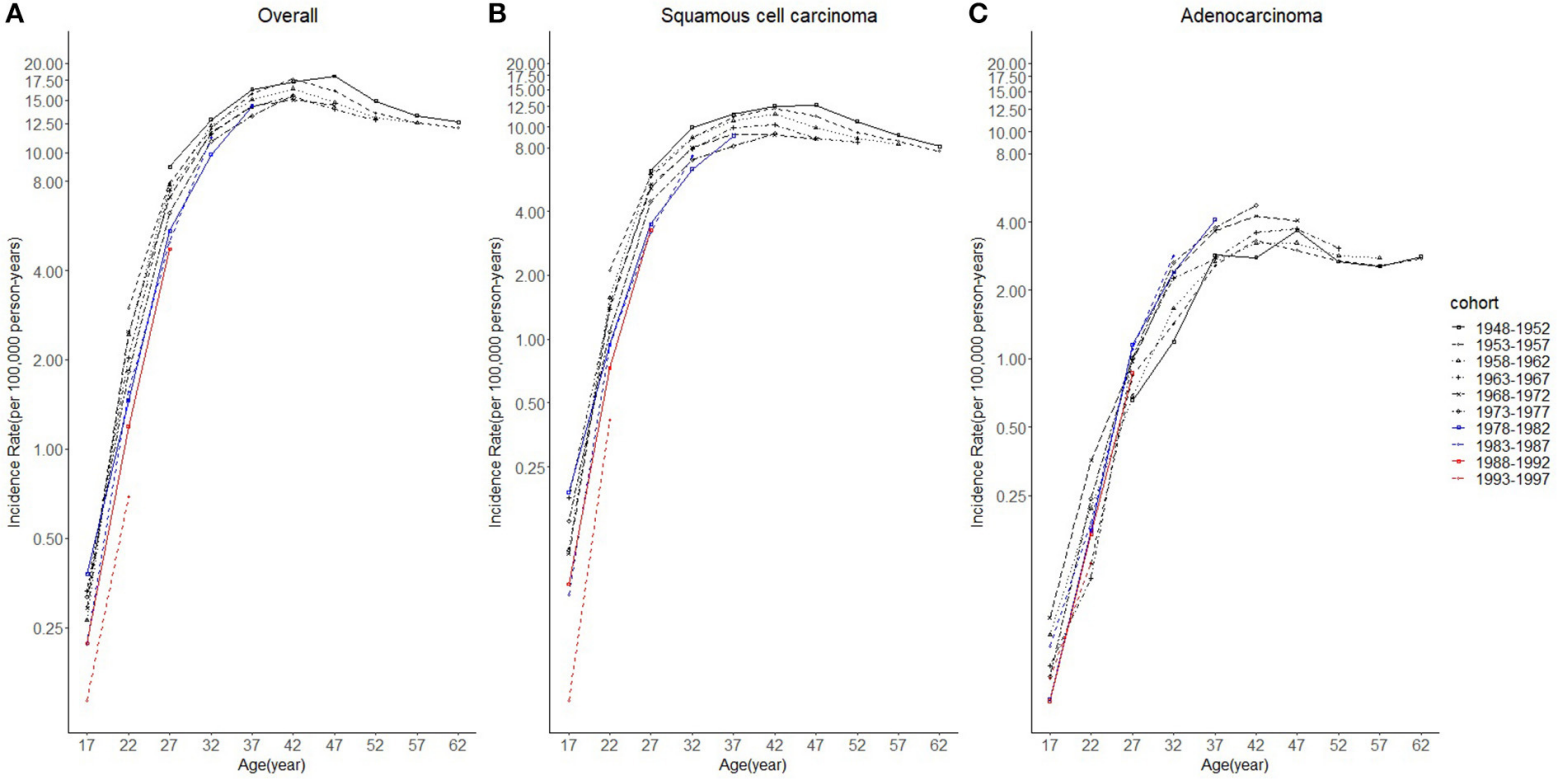
The log-scale age trends in cervical cancer incidence rates (1975–2019) among females in 1948–1997 birth cohorts by age and histology, United States. **(A)** Log-scale age trends of all histopathologic subtypes combined. **(B)** Log-scale age trends of squamous cell carcinoma (SCC). **(C)** Log-scale age trends of adenocarcinoma (AC). All data are expressed as the rate per 100,000.

Considering the few cohort groups of females aged 20–44, females aged 15–64 years were included to analyze the incidence trends by birth cohort group (Figure 4). For SCC, AC, and overall cervical cancer, the incidence rates in the 1988–1997 birth cohorts were lower than those in the 1978–1987 birth cohorts. In particular, for SCC, the 1978–1997 cohort groups had a lower incidence than the 1948–1977 cohorts. By contrast, for cervical AC the incidence rates in the 1978–1997 cohorts were comparable to those of birth cohorts of earlier years. However, the log-scale curves showed a significantly lower rate of incidence in the 1993–1997 birth cohort than in other cohorts (Figure 5).

**Figure 6:**
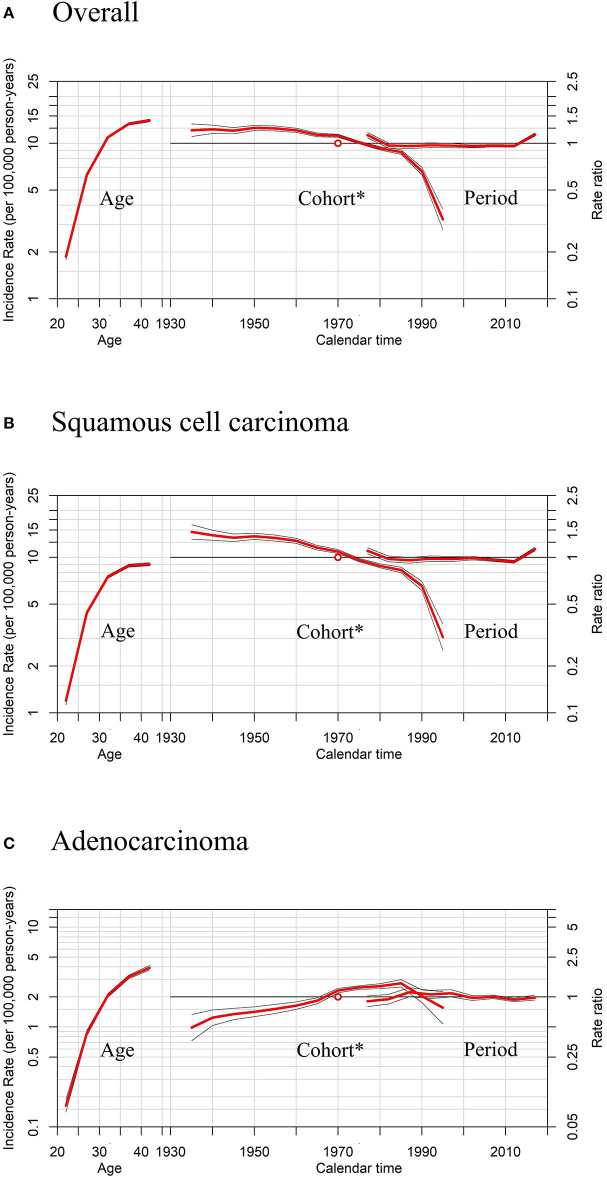
Age, period, and cohort* effects in cervical cancer incidence rates among females aged 20 to 44 years by histology, United States. **(A)** Effects of all histopathologic subtypes combined. **(B)** Effects of squamous cell carcinoma (SCC). **(C)** Effects of adenocarcinoma (AC). * Each plot's horizontal axis is divided into two parts: age, ranging from 20–44 years (left), and calendar time, ranging from 1935–2020 (right). Each plot contains two vertical axes: cervical cancer incidence per 100,000 person-years (left) and rate ratios (right), and three sets of curves: age effects, with corresponding 95% confidence intervals (left); cohort effects, with corresponding 95% confidence intervals (middle); and period effects, with corresponding 95% confidence intervals (right). In cohort effects, the slope of the period was constrained to be 0 to analyze the cohort effect, and the empty red circle represents the reference cohort of 1970 (median date of birth among cervical cancer cases).

APC models indicated the cohort effects and period effects in overall cervical cancer, SCC, and cervical AC incidences (Table 4). Significant improvements in model fitting were found when drift (i.e., the overall linear trend in cervical cancer incidence), period, and cohort effects were added (*P* < 0.05). The best-fitted model included all three effects (age, period, cohort effects), demonstrated by the lowest residual deviance, for overall, SCC, and AC (residual deviance: 57.50, 58.70, 38.82). Females born between 1970 and 1985 had slightly lower cervical cancer incidence than females born in earlier years, while females born after 1985 had visibly lower cervical cancer incidence than those born earlier, as shown in Figures 6A,B. For AC of the cervix, females born between 1960 and 1970 had notably higher cervical cancer incidence than those born earlier, as shown in Figure 6C. Finally, females born between 1970 and 1985 and those born after 1985 experienced a slight increase and a sharp decrease in cervical cancer incidence, respectively, compared to cohorts born earlier.

**Table 4:** Age-period-cohort models for cervical cancer incidence among 20–44-year group females in US, by histological subtype, 1975–2019.

	**Goodness of fit**		**Model comparison**					***EAPC* (95%CI)**
	**Residual df**	**Residual deviance**	**Model comparison**	**Interpretation**	**Change in df**	**Change in deviance**	***p*-value**	
Overall								−0.7^b^ (−1.1, −0.3)
1. Age	40	820.51						
2. Age-drift	39	395.68	2 vs. 1	Trend (drift)	1	424.83	<0.001^a^	
3. Age-cohort	28	223.35	3 vs. 2	Non-linear cohort effect	11	172.33	<0.001^a^	
4. Age-period	32	331.10	4 vs. 2	Non-linear period effect	7	64.58	<0.001^a^	
5. Age-period-cohort	21	57.50	5 vs. 3	Period effect adjusted for cohort	7	165.85	<0.001^a^	
			5 vs. 4	Cohort effect adjusted for period	11	273.60	<0.001^a^	
SCC								−1.0^b^ (−1.4, −0.5)
1. Age	40	1062.65						
2. Age-drift	39	277.28	2 vs. 1	Trend (drift)	1	785.37	<0.001^a^	
3. Age-cohort	28	180.20	3 vs. 2	Non-linear cohort effect	11	97.08	<0.001^a^	
4. Age-period	32	193.82	4 vs. 2	Non-linear period effect	7	83.46	<0.001^a^	
5. Age-period-cohort	21	58.70	5 vs. 3	Period effect adjusted for cohort	7	121.50	<0.001^a^	
			5 vs. 4	Cohort effect adjusted for period	11	135.12	<0.001^a^	
AC								2.6^b^ (0.7, 4.6)
1. Age	40	531.13						
2. Age-drift	39	148.21	2 vs. 1	Trend (drift)	1	382.92	<0.001^a^	
3. Age-cohort	28	56.66	3 vs. 2	Non-linear cohort effect	11	91.55	<0.001^a^	
4. Age-period	32	130.88	4 vs. 2	Non-linear period effect	7	17.33	0.015^a^	
5. Age-period-cohort	21	38.82	5 vs. 3	Period effect adjusted for cohort	7	17.84	0.013^a^	
			5 vs. 4	Cohort effect adjusted for period	11	92.06	<0.001^a^	

CI, confidence interval; df, degrees of freedom; EAPC, estimated annual percent change; SCC, cervical squamous cell carcinoma; AC, cervical adenocarcinoma.

a Indicates significant improvements in model fitting were found when drift, period, and cohort effects had been added at the alpha = 0.05 level.

b Indicates that the EAPC was significantly different from zero at the alpha = 0.05 level.

**Figure 7:**
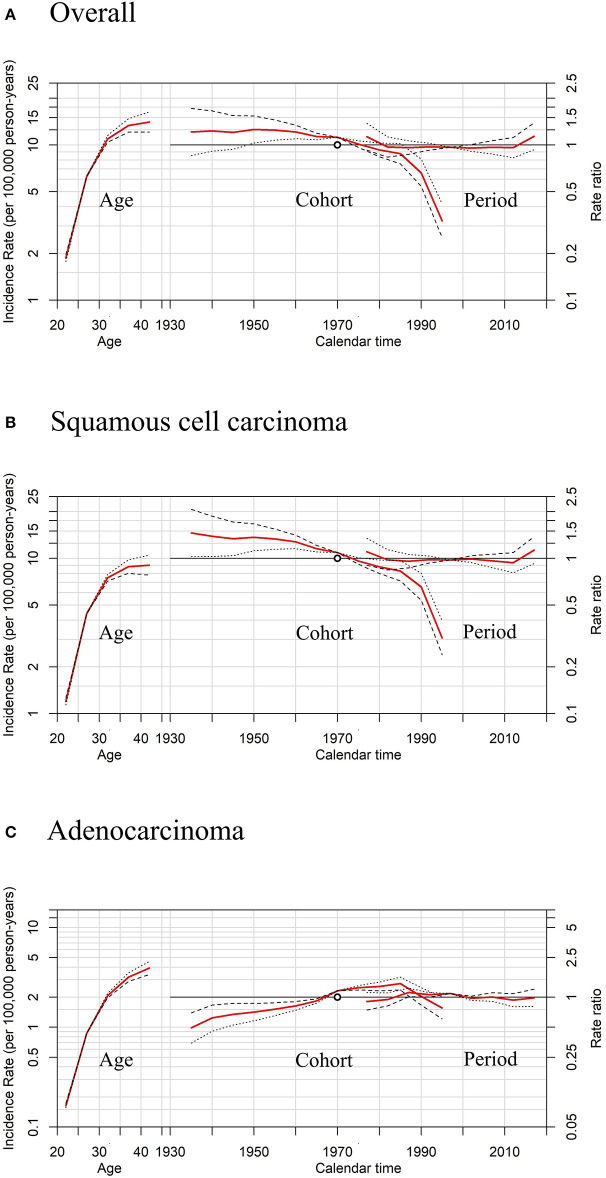
Sensitivity analyses of age-period-cohort model in cervical cancer incidence rates among females aged 20 to 44 years by histology, United States. **(A)** Results of all histopathologic subtypes combined. **(B)** Results of squamous cell carcinoma (SCC). **(C)** Results of adenocarcinoma (AC).The slope of the period effect was assumed to be β = 0 (solid lines), −0.01 (dotted lines), and +0.01 (dashed lines) to analyze the cohort effect. The scale on the left axis is for age effect. The scale for the cohort and period effects is on the right axis. The empty circle represents the reference cohort of 1970 (median date of birth among cervical cancer cases).

Moreover, sensitivity analyses for APC models using different slopes of the period showed that regardless of the period effects, the trends of cohort effects remained the same for both SCC and AC (Figure 7), similar to those in Figure 6.

**Figure 8:**
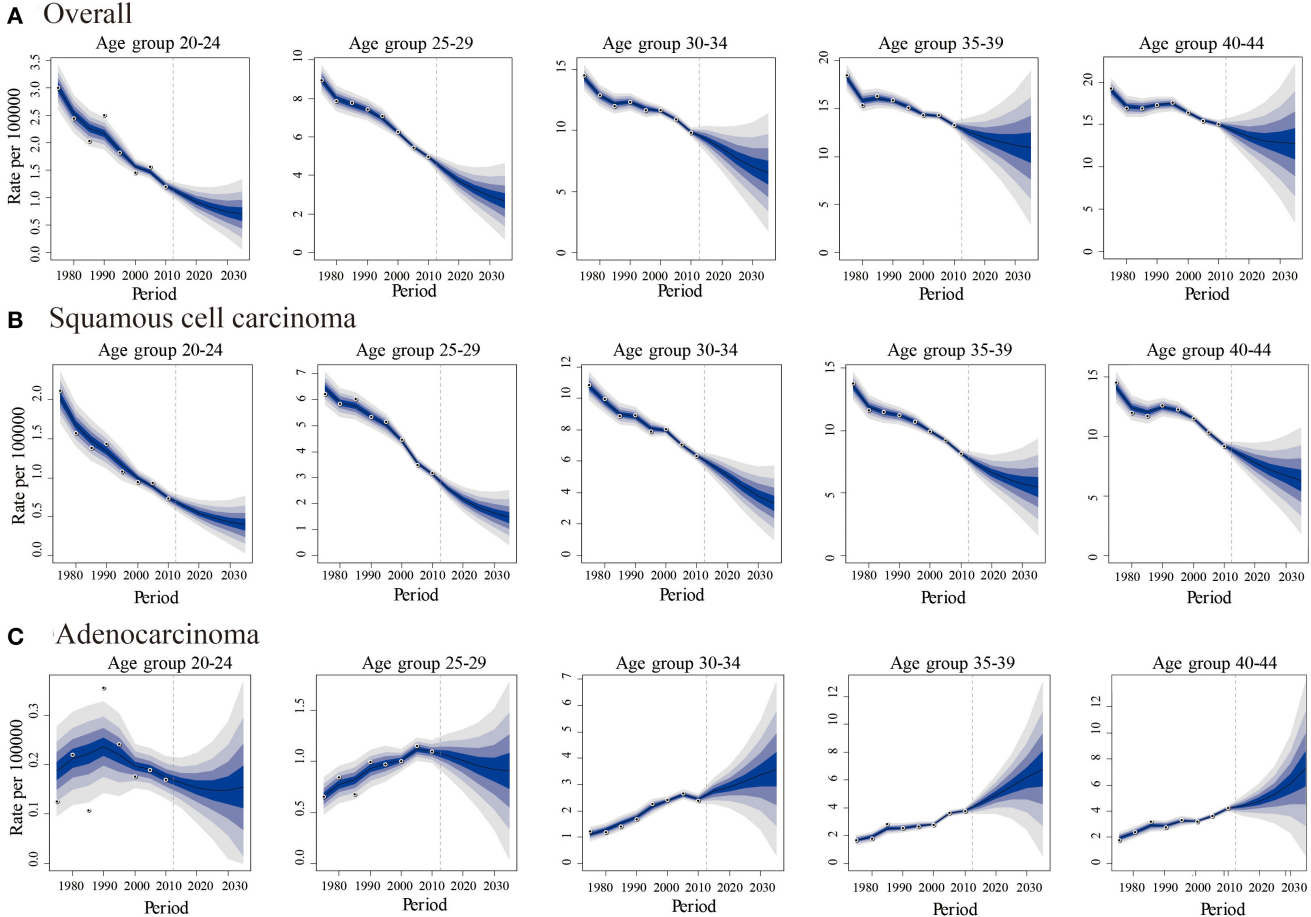
Predicted trends in cervical cancer incidence rates (2015-2039) among females aged 20 to 44 years by age and histology, United States. **(A)** Predicted trends of all histopathologic subtypes combined. **(B)** Predicted trends of squamous cell carcinoma (SCC) **(C)** Predicted trends of adenocarcinoma (AC). All data are expressed as the rate per 100,000. The fan represents the predictive distribution between the 5% and 95% quantiles. Each shaded band represents an additional 10% quantile. The solid line shows the predictive mean, and the white dot represents the observed incidence. The vertical dashed line indicates 2014, when prediction started.

The predicted new cases of cervical cancer for 2035–2039 among 20–44 years old were 19 801, 10 029, 11 068 for overall, SCC and AC, respectively. Predicted incidence cases and rates for SCC, AC, and all histologic subtypes combined by age group between the ages of 20 and 44 from 2015 to 2039 are presented in Tables 5, 6. According to our projection, incidence rates of overall cervical cancer and SCC of the cervix might continue to decrease throughout 2015–2039 in each age group. Of note, the declining trend in the 35–39 and 40–44 age groups gradually slowed down (Figures 8A,B). As for AC of the cervix, the incidence in females aged 20–29 years took on a declining trend. In contrast, the predicted incidence among those aged 30–44 years seems to increase, especially for those in 35–39 and 40–44 age groups (Figure 8C).

**Table 5:** Projected number of new cases of cervical cancer among females aged 20–44 years by age, 2015–2039, United States.

**Period**	**Overall**					**SCC**					**AC**				
	**20−24 y**	**25–29 y**	**30–34 y**	**35–39 y**	**40–44 y**	**20–24 y**	**25–29 y**	**30–34 y**	**35–39 y**	**40–44 y**	**20–24 y**	**25–29 y**	**30–34 y**	**35–39 y**	**40–44 y**
2015–2019	578	2,417	5,016	6,514	7,076	345	1,450	3,111	3,809	4,209	88	598	1,489	2,242	2,200
2020–2024	496	2,140	4,816	6,538	6,977	293	1,244	2,874	3,627	3,996	82	581	1,656	2,699	2,445
2025–2029	441	1,822	4,565	6,652	7,238	257	1,037	2,597	3,540	3,974	79	535	1,844	3,209	2,910
2030–2034	403	1,646	4,055	6,704	7,473	233	918	2,181	3,459	3,913	80	517	1,934	3,704	3,546
2035–2039	371	1,512	3,794	6,392	7,732	212	830	1,938	3,215	3,834	83	516	2,081	3,990	4,398

SCC, cervical squamous cell carcinoma; AC, cervical adenocarcinoma.

**Table 6:** Projected incidence rates (per 100,000 person-years) of cervical cancer among females aged 20–44 years by age, 2015–2039, United States.

**Period**	**Overall**					**SCC**					**AC**				
	**20–24 y**	**25–29 y**	**30–34 y**	**35–39 y**	**40–44 y**	**20–24 y**	**25–29 y**	**30–34 y**	**35–39 y**	**40–44 y**	**20–24 y**	**25–29 y**	**30–34 y**	**35–39 y**	**40–44 y**
2015–2019	1.054	4.276	9.237	12.483	14.211	0.628	2.564	5.725	7.296	8.449	0.159	1.051	2.726	4.277	4.392
2020–2024	0.906	3.678	8.394	11.812	13.428	0.533	2.136	5.006	6.549	7.688	0.146	0.989	2.860	4.838	4.662
2025–2029	0.780	3.158	7.487	11.185	12.705	0.452	1.797	4.261	5.954	6.979	0.133	0.911	2.982	5.323	5.034
2030–2034	0.671	2.743	6.595	10.450	12.022	0.384	1.531	3.553	5.402	6.306	0.122	0.836	3.076	5.652	5.580
2035–2039	0.577	2.360	5.811	9.676	11.308	0.326	1.300	2.983	4.889	5.634	0.112	0.766	3.076	5.834	6.212

SCC, cervical squamous cell carcinoma; AC, cervical adenocarcinoma.

## Discussion

In this study, we calculated the temporal and age-period-cohort effects and future trends of cervical cancer incidence in the US, the results of which provide supporting evidence for the effectiveness of the HPV vaccine in preventing cervical cancer, regardless of the histological subtype.

Reasons for the changing trends of cervical cancer incidence might be multi-faceted. The increasing overall pattern could be due to *in-utero* exposure to maternal diethylstilbestrol (DES) (37), obesity (38), and long-term use of hormonal contraceptives (39). However, the overall increased incidence of cervical cancer could be due to two factors. First, from WHO classification of female genital tumors (5th Edition) (40), the vast majority (>90–95%) of squamous cell carcinoma of the cervix is caused by high-risk HPV genotypes, while HPV-associated adenocarcinoma of the cervix accounts for ~75% of all cervical adenocarcinomas. HPV 16 and 18 together account for 95% of HPV-associated cervical adenocarcinoma (41, 42). As the majority of cervical cancer were caused by HPV infections (3), HPV vaccination might contribute to reducing the incidence. Second, it could be the impact of the current screening policy targeted at cervical cancer (43). Since the 1960s and 1990s, the Papanicolaou (Pap) test and Thinprep cytologic test (TCT) were widely applied, successively (43). Thereafter, Pap/HPV DNA co-testing and primary testing were included in screening guidelines in 2012 and 2018 (44). For the Pap and TCT tests, sensitivity is superior for cervical SCC than for AC (45, 46). Since less is known about the natural history of AC of the cervix, the impact of Pap tests on its incidence is unclear (47). Chronologically increasing trends in the incidence rate of AC in the US from 1975 to 2013 resemble those of other non-screen-detected HPV-associated cancers, such as oropharyngeal and anal cancer (48). Therefore, the incidence trends and cohort effects for cervical AC, rather than SCC, could more likely reflect the impact of HPV vaccines.

The quadrivalent human papillomavirus vaccine was first recommended by the ACIP in the US in 2006 (5). The vaccine is recommended for girls 11–12 years old and can also be administered to girls as young as nine. Catch-up vaccination is recommended for females aged 13–26 years who were not previously vaccinated (5). In the US, the coverage of HPV vaccine initiations (received ≥1 dose) was 25.1, 53.8, 69.9, and 77.1% in 2007, 2012, 2018, and 2020, respectively, among adolescent females aged 13–17. The coverage among females aged 19–26 years increased from 20.7% in 2010 to 52.8% in 2018 (9–11); only 2.7% of adults aged ≥34 in 2017 had been vaccinated (38). Our results revealed that the temporal trends of AC incidence rate have been increasing throughout 1975–2019, where trends in the 20–29 age groups remained relatively stable, whereas the incidence displayed an increasing trend in the 30–34, 35–39, and 40–44 age groups, particularly in the latter two groups. Compared to the 35–44 age groups, the 20–29-year age groups who received HPV vaccines exhibited lower incidences of cervical AC. Since females 35–39, 40–44 years of age in 2019 were 23–27, 28–32 years of age in 2007 and 28–32, 33–37 years of age in 2012, they would not have been within the recommended window for catch-up HPV vaccination when first approved, and either not in the age intervals when the coverage of HPV vaccination reached over 50%. Similarly, according to the projected trends from Bayesian APC analyses, AC incidence of females aged 20–30 will decline, but increase among those aged 31–40, especially in the 35–44 age group.

**Figure 9:**
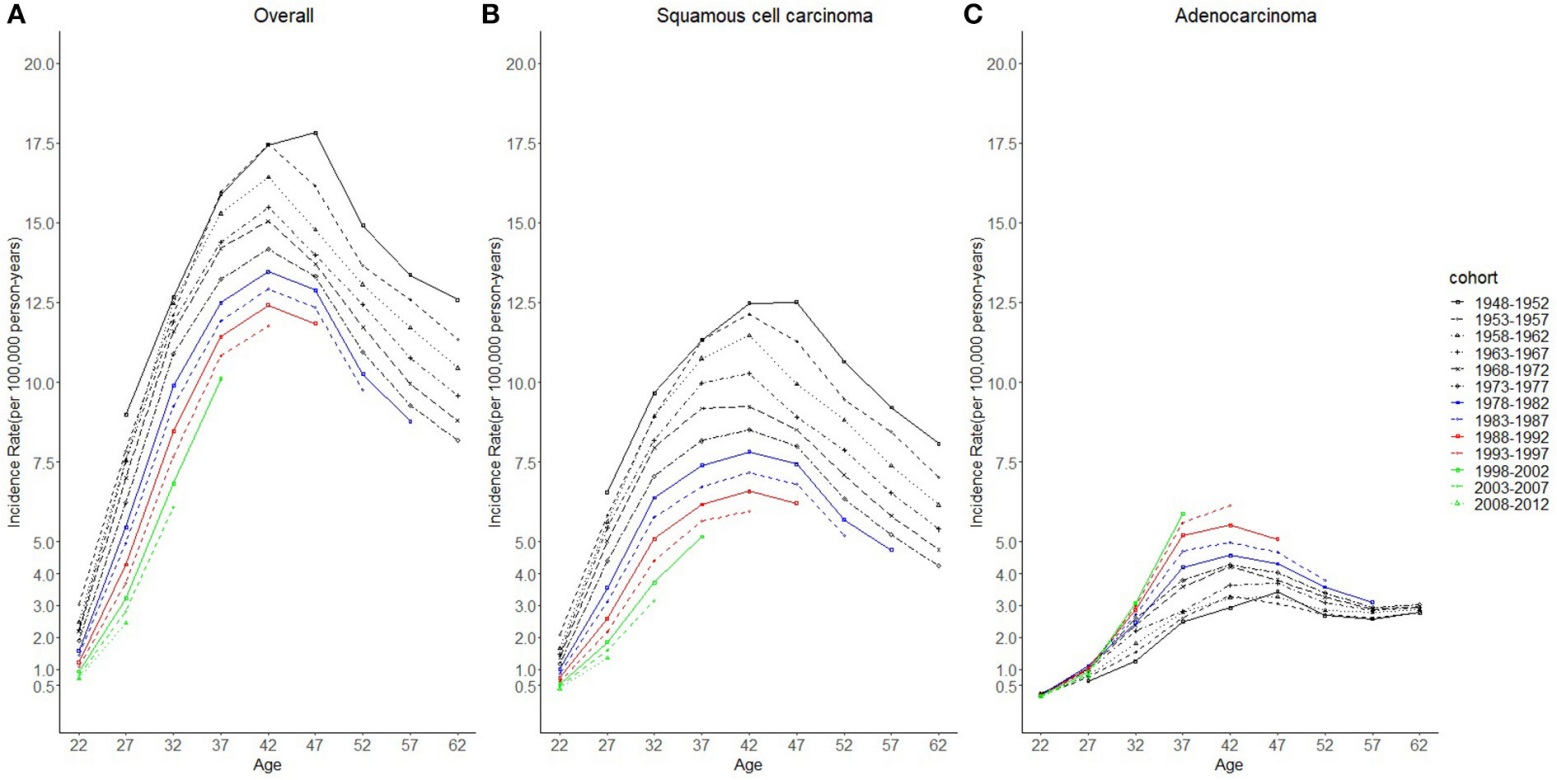
Predicted age trends in cervical cancer incidence rates among females in 1948–1997 birth cohorts by cohort and histology, United States. **(A)** Predicted age trends of all histopathologic subtypes combined. **(B)** Predicted age trends of squamous cell carcinoma (SCC). **(C)** Predicted age trends of adenocarcinoma (AC). All data are expressed as the rate per 100,000.

Specifically, from the results of incidence trends by birth cohorts, regardless of the histological subtype, cervical cancer incidence rates in the 1988–1997 birth cohorts when there was greater coverage of HPV vaccination, were lower than those of the 1978–1987 birth cohorts. For AC of the cervix, considering increasing incidence, the incidence in the 1978–1997 cohorts was comparable to that from the 1948–1977 cohorts. The incidence in the 1993–1997 birth cohort with the highest vaccination coverage displayed a markedly slower growth rate than others. The predicted rates by birth cohort groups from BAPC analyses (Figure 9) showed that regarding SCC of the cervix, the younger the cohort population, the lower the incidence. For AC, after 30 years old, the younger cohorts exhibited higher incidence trends than older ones. Although the reason might be the increasing incidence of AC, the differences of predicted trends among 1998–2012 birth cohort, 1988–1997 birth cohort, and 1948–1987 birth cohort might be misleading. Because, based on the current risk factors, projections obtained through BAPC cannot foresee the changes in risk factors or new interventions in the future (33); thus, BAPC did not account for the potentially increasing coverage of HPV vaccination for 1998–2012 and 1988–1997 birth cohorts from 2015 to 2039.

The cohort effects for cervical AC also suggested the effectiveness of HPV vaccination. AC incidence in females born after 1985 exhibited a sharp decline and was less than or equal to that of those 26 years old in 2011. These findings were consistent with the cohort effects of cervical intraepithelial neoplasia grades 2 or 3 and AC *in situ* (collectively, CIN2+) incidence among TennCare-enrolled females in the state of Tennessee, US (22). Furthermore, the cohort effects of cervical SCC displaying a decreasing trend in the 1985–1997 birth cohorts were likely due to generational differences in HPV vaccine eligibility and vaccination behaviors.

National guidelines currently recommend that women undergo Pap testing at 3-year intervals starting at 21 years old and plus HPV DNA testing from the age of 30 years, regardless of HPV vaccination status (49–51). The screening policy has changed several times during the study period: the interval of screening, not recommended for young females aged < 21 years, was prolonged (50–53). In the US, the screening coverage rate declined modestly in recent years (54, 55), especially among the 20–29-year age group, consistent with the trending of overall incidence of cervical cancer and SCC of the cervix. Nevertheless, the screening uptake in the 30–39 age group showed an increasing trend during 2013–2015 (54), which could have contributed to the increasing incidence of overall cervical cancer and SCC of the cervix among the same age groups during 2012–2019. The period effects of overall cervical cancer and SCC were significant, which implied that the influences were caused by changes in the screening policy. Meanwhile, the period trends in AC exhibited a mild increase, and the period effects for AC were significant (*P* = 0.013). The potential reason could be that the Pap/HPV DNA co-test was sensitive to AC of the cervix.

Although screening with subsequent treatment for precancerous cervical lesions has significantly reduced the incidence of cervical cancer (56), not all females receive screening or follow-up treatment, and cervical cancer remains a considerable cause of mortality (57). Besides, based on our BAPC analyses, the incidence of cervical AC is likely to continue to increase from 2015 to 2039. The future disease burden of AC may be 11 068 cases among females aged 20–44 years in 2035–2039, while it might be 6 617 cases in 2015–2019. The burden of overall cervical cancer in 2035–2039 (19 801 cases) appears to resemble that in 2015–2019 (21 602 cases). Therefore, expanded HPV vaccine coverage may be an effective approach to achieving general reductions in cervical cancer over the coming years. Our projection of incidence rates during the 2015–2019 period was comparable with that from the SEER program, indicating that our predictions could be considered reliable.

Our study has some limitations. The data in our study did not include all individuals in the US. However, by analyzing the Seer 9, Seer 13, and Seer 22 program combined data, which covered nearly 47.9% of the US population, our research has an advantage over other studies that solely used the Seer 9 program (which covered 9.4% of the US population). Besides, we used the latest data from the Seer 22 program, which was released on April 15, 2022. Moreover, as an ecologic study, we were unable to examine individual-level potential confounders and vaccination data, but instead were able to use the age-period-cohort model to explain most confounding factors such as age effects and period effects and provide a methodologically acceptable design for studying the intervention effects of HPV vaccination. Our study was the first to comprehensively examine the birth-cohort effects before and after the HPV vaccination had been introduced in recent decades.

## Conclusions

The cervical AC incidence increased from 1975 to 2019; however, this increase involved only those who did not receive the HPV vaccine, while the incidence was stable in the vaccinated. The projected trends showed similar results whereby vaccinated females will exhibit a decline in cervical AC incidence rate, whereas the unvaccinated will display an increased rate. The birth cohort effects of SCC, AC, and all histologic subtypes combined also provide evidence for the effectiveness of the HPV vaccine in preventing cervical cancer.

## Data availability statement

Publicly available datasets were analyzed in this study. This data can be found here: the National Cancer Institute's (NCI's) Surveillance, Epidemiology, and End Results (SEER) Program 9+13+22.

## Author contributions

JP: conceptualization, methodology [software], data curation, formal analysis, writing—original draft, and writing—review and editing. TS: methodology [software], data curation and analysis, validation, and writing—review. CW: data analysis and writing—review and editing. ML and MX: methodology [software], data analysis, and writing—review. CZ: conceptualization, methodology, formal analysis, validation, project administration, and writing—review and editing. MJ: conceptualization, methodology, formal analysis, validation, supervision, and writing—review and editing. All authors contributed to the article and approved the submitted version.
